# Systematic review on HIV situation in Addis Ababa, Ethiopia

**DOI:** 10.1186/s12889-019-7885-8

**Published:** 2019-11-21

**Authors:** Melaku Adal

**Affiliations:** Microbial, Cellular and Molecular Biology Department, Addis Ababa University, Addis Ababa, Ethiopia

**Keywords:** HIV/AIDS, Hotspot, Predisposing factors, Interventions

## Abstract

**Background:**

HIV prevalence in the Addis Ababa is still higher in key and priority populations. Therefore, this systematic review was carried out aiming in determining the prevalence of HIV and predisposing risk factors, identification of hotspot areas, key and priority populations, availability and utilization of services, and challenges and gaps to be addressed for prevention and control of HIV epidemic in Addis Ababa.

**Methods:**

The documents relevant to address the objectives were collected from online databases Google scholar and PubMed for published works. In addition, unpublished survey and surveillance reports, performance reports and project assessment findings, and mapping results were collected from partner organizations working on HIV/AIDS prevention and control.

**Results:**

It appears that the HIV prevalence stabilizes, but varies along areas and socio-demographic groups. The most common hot spots in Addis Ababa are areas where bars, groceries, pensions, guest houses, hotels, brothels, massage houses, khat houses, shisha houses, night clubs, drinking establishments and tourist frequented settings are concentrated. The recognized key population (KP) is the female sex workers (FSWs). There is sexual mixing of key and priority populations (KPPs) with the general population. There are various behavioural, biological and socio-economic predisposing risk factors that drive HIV epidemic, and respective behavioural, biomedical and structural intervention measures are identified in the presence of gaps and challenges to address the problem.

**Conclusions:**

HIV prevalence in Addis Ababa seems stabilized. However, it varies along different groups of the population. There are many behavioural, biological and socio-economic factors that predisposed to HIV/AIDS. Weak monitoring of the quality of interventions, limited linkage of positive clients, lost to follow up, financial shortage, limited man-power and coordination, data quality and gaps in use of program data or research findings are some of the gaps and challenges. Therefore, prevention and control measures using behavioural, structural and biomedical interventions through filling of gaps and tackle challenges should be strengthened in order to prevent and control HIV transmission.

## Background

According to HIV related estimates and projections for Ethiopia [1], there are 610,335 people living with HIV (PLHIV) with estimated adult HIV prevalence being 0.96%. The Ethiopian demographic and health survey (EDHS) 2016 report [2] shows Gambella region (4.8%) and Addis Ababa (3.4%) to have the highest HIV prevalence rates while Somali (< 0.1%), and Southern Nations, Nationalities and peoples (SNNP, 0.4%) regional states have the lowest. The adult (15–59) HIV prevalence in Ethiopia is 0.9%, with varying burden by sex, age, and other demographic characteristics, across sub-regions and population groups. The urban HIV prevalence (2.9%) is seven times higher than the prevalence in rural settings (0.4%), women (1.2%) having twice higher HIV prevalence than men (0.6%) [2].

The Ethiopian population based HIV impact assessment (EPHIA) [3] showed that prevalence of HIV is 3.0% in urban settings [3]. This relatively high prevalence of HIV in Addis Ababa initiated us to look into the magnitude of the HIV prevalence, who and why they are affected, the availability and utilization of services for the most affected groups, and the gaps and challenges to address the problem. Therefore, this systematic review was carried out aiming in determining the prevalence of HIV (1), predisposing risk factors (2), identification of hotspot areas (3), key and priority populations (4), availability and utilization of services, and challenges and gaps to be addressed for prevention and control (5) of HIV epidemic in Addis Ababa.

## Methods

### Protocol and registration

This study has been designed and reported according to the Preferred Reporting Items for Systematic Reviews and Meta-Analysis (PRISMA) tool [4]. Analytic methods and inclusion criteria were specified and documented in advance.

### Eligibility criteria

Studies carried out including Addis Ababa as study site from 2005 to April 2019, written in English and address HIV prevalence; predisposing behavioural, biological and socio-demographic risk factors; identified hotspot areas; identifications of most-at-risk populations (MARPS) such as key and priority populations; and intervention strategies such as behavioural, biomedical and structural intervention strategies and availability of services; and identifying gaps that could be challenges and opportunities for prevention and control of HIV/AIDS were included and analyzed following the above category.

### Information sources

The documents relevant to be reviewed to address Addis Ababa were collected from online databases Google scholar and Pubmed for published work. In addition to published works, unpublished survey and surveillance reports, performance reports and project assessment findings, and mapping results were collected from Federal Ministry of Health (FMOH), Ethiopian Public Institute (EPHI), Ethiopian Public Health Association (EPHA), Organization for Social Services, Health and Development (OSSHD), Federal HIV/AIDS Prevention and Control Office (FHAPCO), Addis Ababa HIV/AIDS prevention and Control Office (AAHAPCO), Addis Ababa Health Bureau (AAHB), Population Services International/Ethiopia (PSI/E), American Centre for Disease Control and Prevention (CDC) and World Health Organization (WHO).

### Search

The following terms and phrases were used as needed: HIV, AIDS, prevalence, highly active antiretroviral therapy, HAART, antiretroviral therapy, ART, compliance, adherence, resistance, predisposing factors, behavioural, biological, socioeconomic, most-at-risk populations (MARPs), khat, alcohol, drug use, knowledge, attitude, practice, KAP, and behavioural change; condom use, abstinence, faithfulness, stigma, discrimination, HIV counseling and testing, HCT, voluntary counseling and testing, VCT, prevention of mother to child transmission and PMTCT.

### Study selection, data collection and items

**Fig. 1:**
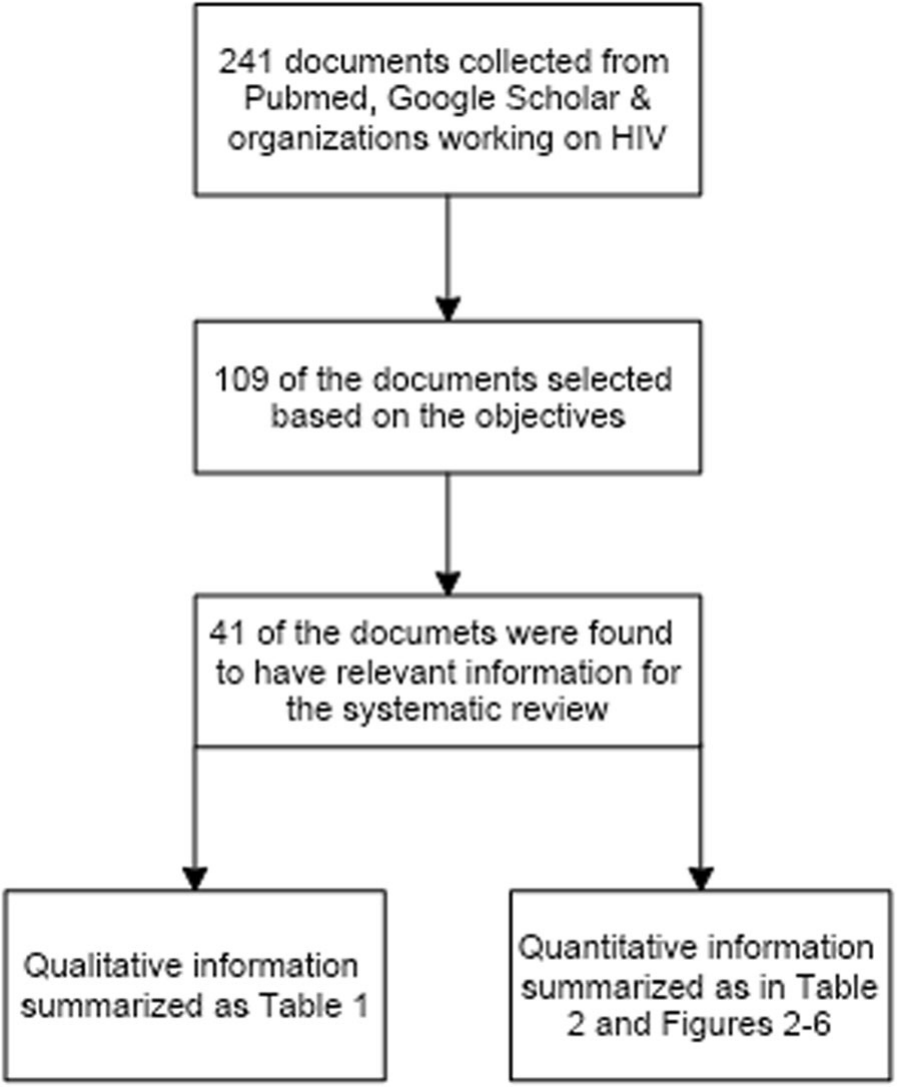
The flow chart used for collection of qualitative and quantitative data

A total of 241 documents were collected, among which only 109 of them were relevant and used for the quick review. The quick review form was developed to collect all necessary information of the source document with full citation, main findings, and to match to specific objectives. After quick review, 41 documents with relevant information were selected and used. Quantitative data were collected from surveys and performance reports. Based on a closer and in-depth review of quantitative data, the raw information was categorized under exclusive thematic areas based on the specific objectives in order to make the data presentation easier (Fig. 1). Furthermore, the quantitative methods were applied in synthesizing of secondary data and present it in tables and graphs.

### Synthesis of results

**Fig. 2:**
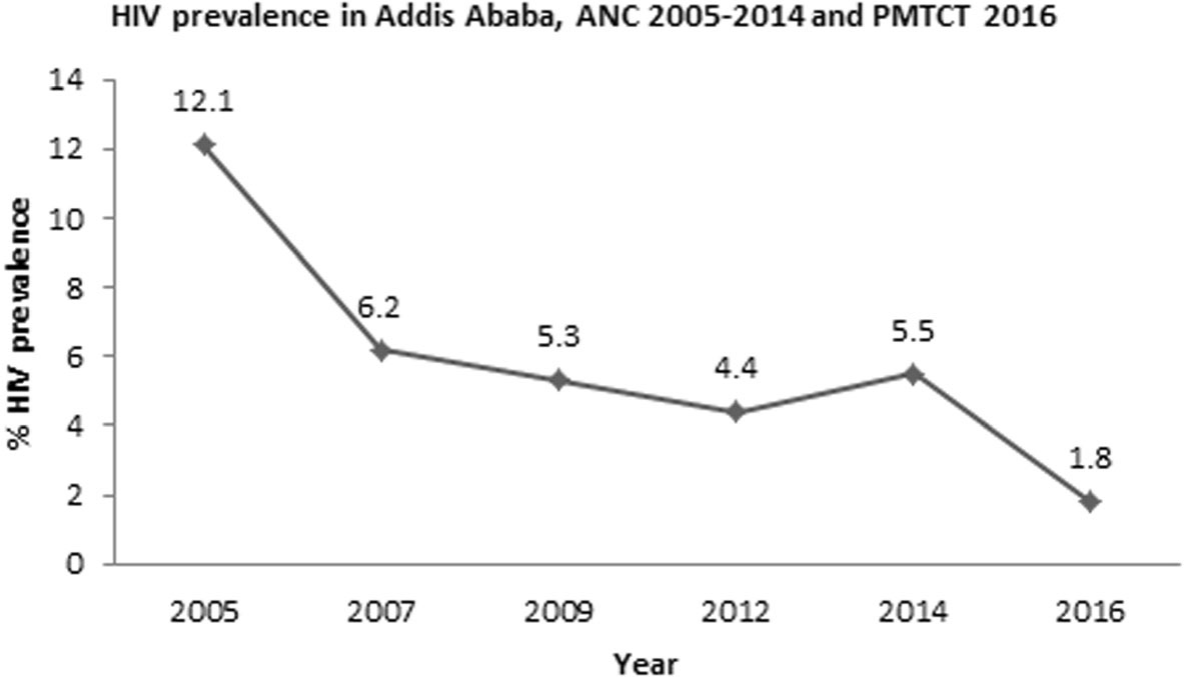
HIV prevalence in Addis Ababa, ANC 2005–2014 and PMTCT 2016 [7–10]

**Fig. 3:**
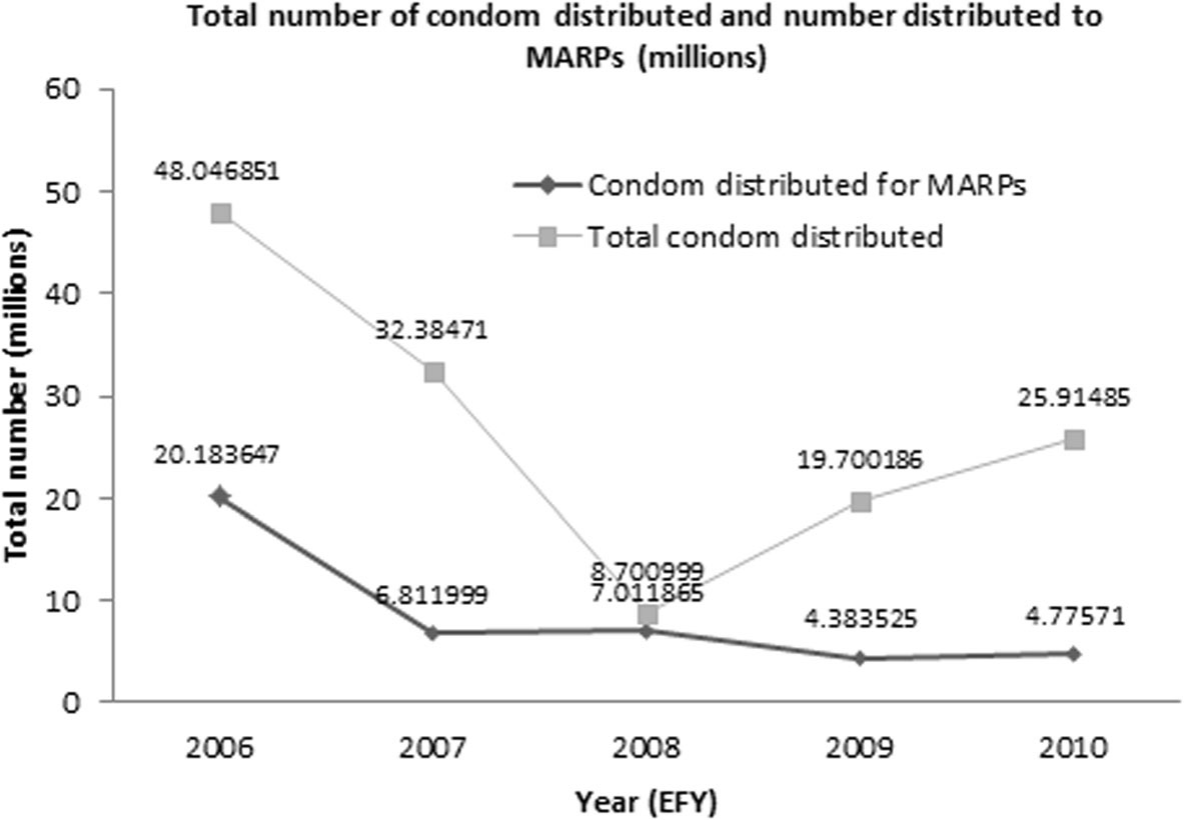
Total condom distributed and condom distributed for MARPs from 2006 to 2010 EFY [11]. EFY = Ethiopian fiscal year

**Fig. 4:**
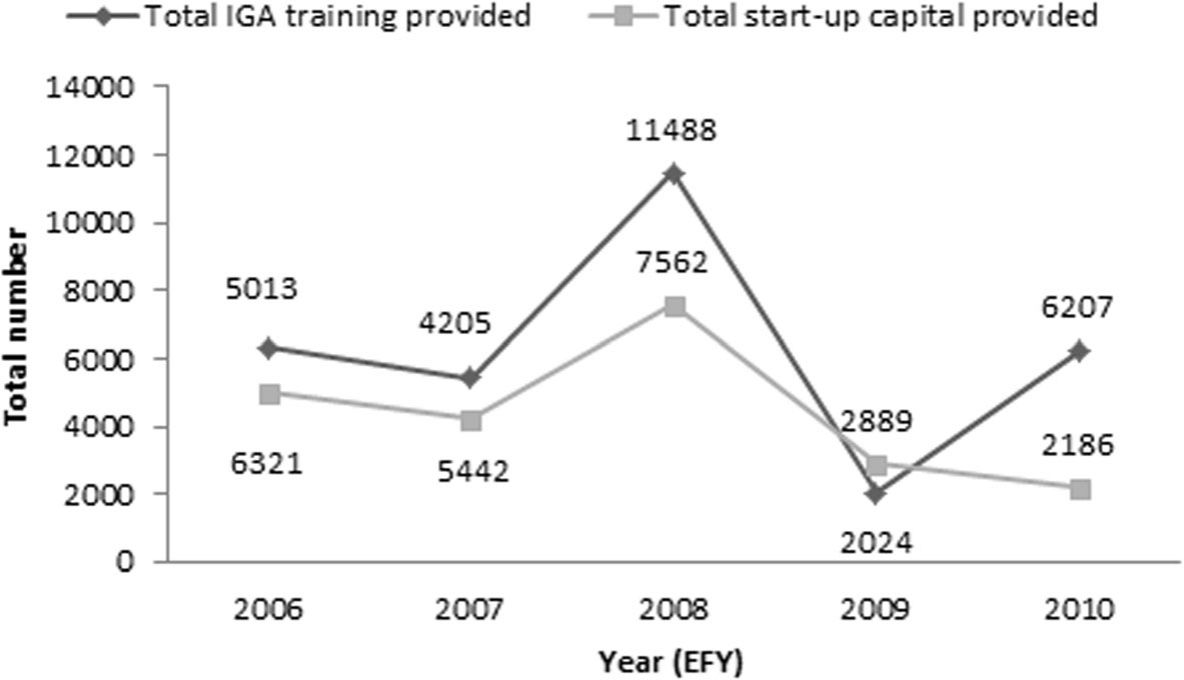
Total number of individuals who were provided IGA training and start-up capital from 2006 to 2010 EFY; IGA = income generating activities [11]. EFY = Ethiopian fiscal year

**Fig. 5:**
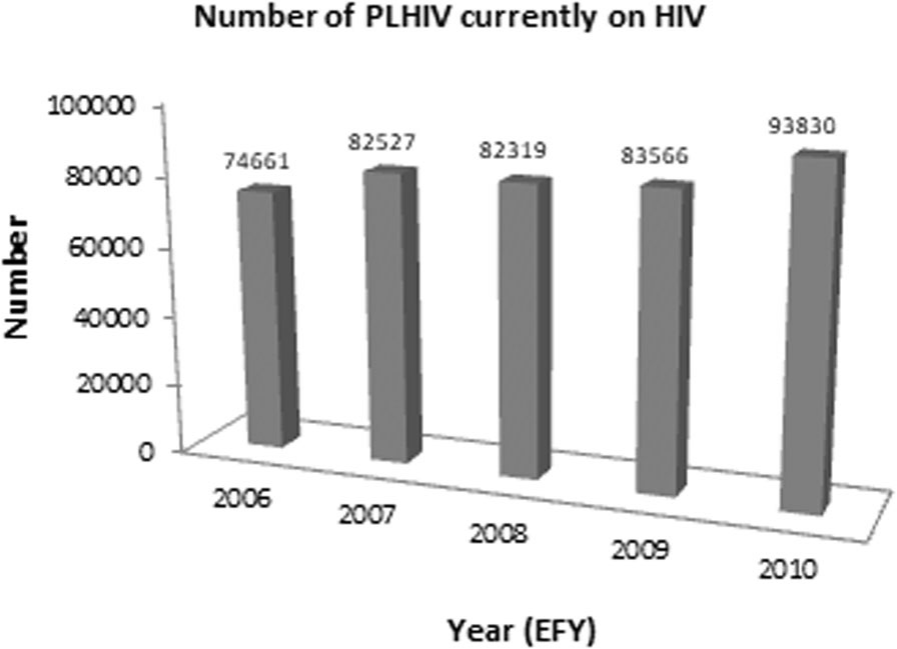
Number of individuals currently on ART (cumulative), 2006–2011 EFY [11]. EFY = Ethiopian fiscal year

**Fig. 6:**
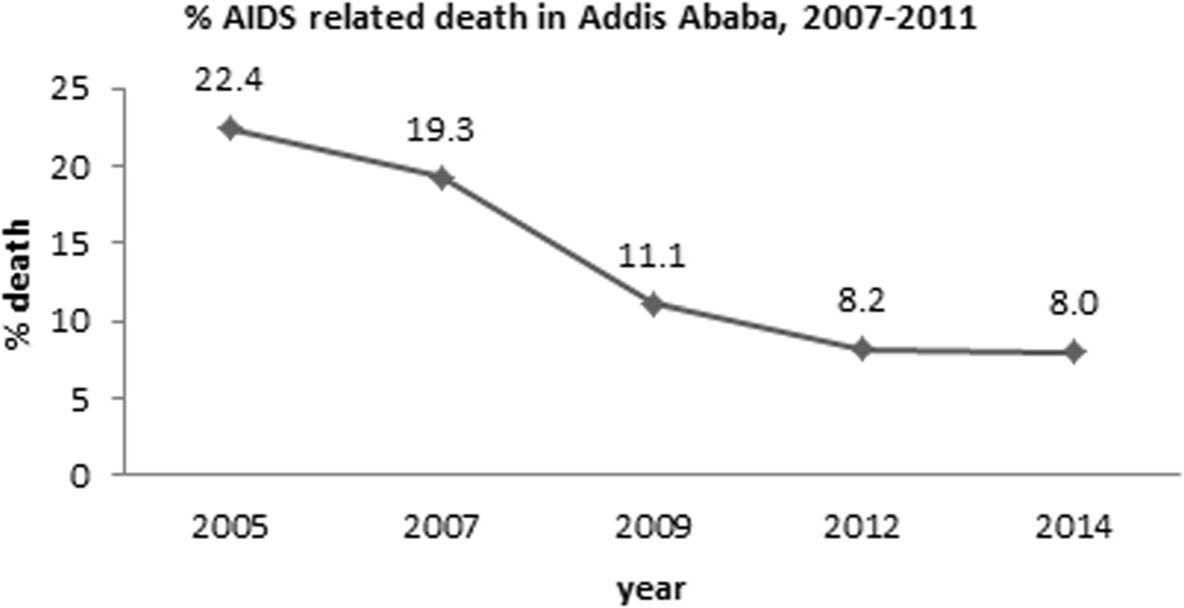
Percentage of AIDS death in Addis Ababa from 2007 to 2011 [12]

The qualitative information are presented as in Table 1 and described in text. In addition, the quantitative information is summarized in Table 2 and Figs. 2, 3, 4, 5, 6.

**Table 1:** Summary information about the source materials used

No.	Documents	Type of document	Key findings	Objectives
1	EPHI, 2018a	Report	• HIV prevalence Table 2 and Figure 1	1
2	CSA and ICF, 2016	Survey	• HIV prevalence 3.4% in Addis Ababa • Men who had sex with non-cohabiting partners is highest in Addis Ababa (26%) than the national average (16%) • The mean number of lifetime sexual partners reported by men in Addis Ababa (5.2%) • Women reported using a condom during last sexual intercourse with non-regular partners 41.8% and men 72.4% • Discordant couples (4.3%)	1, 3
3	EPHI, 2018b	Survey	• HIV prevalence is 3.1% in Addis Ababa • VLS of whole country in urban areas is 70.1% (Female 71.7% and Male 66.8%), varies by age, sex, and region, • Status of the three 90’s in Addis Ababa: 65.2 % for the 1^st^ 90, 63.3 % for the 2^nd^ 90 and 58.2% of all PLHIV	1, 5
4	Moher *et al*., 2015	Article	• PRISMA Statement	-
5	CSA and ORC, 2005	Survey	• HIV prevalence is 4.7% in Addis Ababa	1, 3
6	CSA and ICF, 2011	Survey	• HIV prevalence is 5.2% in Addis Ababa	1, 3
7	EPHI, 2015	Report	• Figure 1 for HIV prevalence	1
8	EPHI, 2011	Report	• Figure 1 for HIV prevalence	1
9	EPHI, 2014	Report	• Figure 1 for HIV prevalence	1
10	EPHI, 2017	Report	• Figure 1 for HIV prevalence	1
11	FHAPCO, 2018	Report	• Behavioural, biomedical and structural interventions • ART coverage is 74.6%; viral load testing coverage ~60% with 87.5% VLS • In Addis Ababa, the total number on ART were 94,240 and 3,616 were newly enrolled; retention at 12 months 87% • Figures 2, 3, 4	1, 5
12	EPHA/CDC (2012)	Report	• Death related to HIV/AIDS in Figure 5	1
13	AAHAPCO, 2017	Synthesis	• Key drivers of the epidemic; hotspot areas; intervention strategies; challenges on intervention	2, 3, 4, 5
14	Lakew *et.al.,* 2015	Article	• 5.7% HIV-positives among mobile workers	1, 4
15	FMOE, 2012	Survey	• low level of knowledge, peer pressure, practices of unsafe sex, the proliferation of addictions (shisha, khat, alcohol) and substance abuse, gender–based violence were driving forces for the spread of the epidemic.	3, 4, 5
16	PSI/E, __	Research brief	• Non-self-identified (NSI) FSWs to supplement their income to support family or the desire for fashion and luxury goods • The main barrier to condom use is higher payment, in addition to intimacy and trust with long-term clients • NSI FSWs felt some polices favor clients and they would be unlikely to get a positive outcome by reporting violence • NSI FSWs may be more likely to experience violence, but less likely to report it given the hidden nature of their work	3
17	Deyessa *et al.,* 2018	Survey	• Male users dominated female users at a ratio of 9:1; 3/4 of the IDUs were below the age of 35 years • The estimated IDUs in Addis Ababa were 4,068 • The majority, 200 (72.5%) of the drug users from Addis Ababa had the habit of reusing needle and syringe • Of the 177 Addis Ababa residents who claimed to have tested for HIV, 70 (39.5%) disclosed as HIV positive	1, 3 ,4
18	Cherie *et al.,* 2012	Article	• Peer pressure is the most important factor associated with risky sexual behavior among school adolescents	3
19	Mirkuzie (2018)	Article	• 2% and 4% of the HIV exposed babies were HIV positive by 6 and 18 months, respectively • No prophylactic ART and mixed feeding were predictors for having an HIV positive antibody test at 18 months	5
20	Klaus *et al.,* 2015	Article	• The barriers to PMTCT completion: hopelessness and carelessness, lack of understanding of the efficacy of ARV, and negative religious influences.	3
21	Endalamaw *et al.,* 2018	Article	• Rural residence, home delivery, no ART prophylaxis and mixed feeding increased the risk of HIV transmission	3
22	Menna *et al.,* 2014	Article	• High knowledge of HIV/AIDS, attitude towards ‘ABC’ rules, being tested for HIV and chewing khat are factors associated with multiple sexual partnerships among secondary school students.	3
23	EPHA *et al.,* 2013	Report	• The estimated HIV prevalence among FSWs in towns was 23.0%,; 4.5% in truck drivers • ~15.5% of drivers have misconceptions about HIV prevention methods • 21 % of drivers accept that once they had unprotected sex with someone, there is no reason to use condoms • Divorced/Separated/Widowed have also high HIV prevalence	1, 3, 4
24	UNODC, 2014	Survey	• HIV prevalence 4.2% in prison settings	1, 4
25	PEPFAR, 2018	Strategic Plan	• There are about 200,000 FSWs in Ethiopia	1, 4
26	PSI/E, 2016	Research brief	• The majority of FSWs (57.5 %) are 24 years and younger, and about 14% are 19 years or younger • > 6% of HIV positive FSWs who started ART reported discontinuation of treatment for more than seven days in the three months prior to the assessment	1, 4, 5
27	Demissie *et al.,* 2018	Article	• The prevalence of HIV among IDUs was 6% • 40% of IDUs reported ever sharing needles; 56% reported sharing other injecting equipment; among HIV-positive IDUs, 60% reported sharing a needle the last time they injected. • Most of the IDUs were males (96%) with a mean age of 26 years.	1, 3, 4
28	FHAPCO, 2018	National roadmap	• Key and priority populations	4
29	FMoH, 2018	Report	• Behavioural, biomedical and structural interventions	5
30	Biadgilign *et al.*, 2011	Article	• Parents refusing to give consent for their children to access HIV testing services (HTS) and ART services	5
31	Gesesew *et al.,* 2016	Article	• Males being away from home, drug addiction, fear of stigma & discrimination, distance from ART clinics, dependent on food supplies, mental problems, HIV negative partners; and baseline CD4 <200 cells/mm3 and WHO clinical stages 3 & 4 were factors of ART discontinuation.	5
32	Gesesew *et al.,* 2017a	Article	• Being rural dweller, illiteracy, marriage, alcohol use, smoking, having mental illness and being bed ridden functional status, having HIV positive partner and being co-infected with TB/HIV were factors for ART discontinuation.	5
33	Gesesew *et al.,* 2017b	Article	• ART discontinued adults were more likely to be females, tuberculosis/HIV co-infected, with immunological failure and no history of HIV testing.	5
34	Bezabhe *et al.,* 2014	Article	• Economic constraints, perceived stigma & discrimination, medication side effects, and dissatisfaction with healthcare services, disclosure of HIV status, social support, responsibility for raising children, improved health on ART, and receiving education and counseling were factors for patients being non-adherent and lost to follow-up	5
35	Tiruneh and Wilson, 2016	Article	• With the introduction of appointment spacing, some patients complain of lack of storage space for the six-month supply of ARTs, poor storage conditions for their medicines, and preference of frequent follow up. Health workers are also concerned about adherence given the less frequent contact of PLHIV with the health services	5
36	PEPFAR, 2016	Operation plan	• Key and priority populations	4
37	FHAPCO, 2014	Strategic plan	• HIV transmission interventions include behavioural, biomedical and structural components. • The plan intends to achieve the three 90 targets by 2020 through targeted social mobilization and HIV testing, linkage to care, quality of HIV treatment, and virtual elimination of MTCT, envisioning ending AIDS by 2030	5
38	Gudina *et al.,* 2017	Article	• Combination ART acheives sustained HIV viral suppression and contributes to improvement in the quality of life; and reductions in mortality, progression to AIDS, opportunistic infections (OIs), hospitalization, and decreased HIV transmission to uninfected persons	5
39	Misgena, 2011	Article	• Challenges related to HAART include lifelong therapy, failed treatment response, optimal time to start treatment and switching regimens, drug interaction, toxicity, cardiovascular risks, drug resistance, lost to follow-up, immune reconstitution inflammatory syndrome (IRIS), early mortality, challenges in viral load testing.	5
40	Bernabas *et al.,* 2017	Article	• Noncompliance to medical instruction and poor adherence fosters emergence of drug resistance. Limited availability of laboratory services such as HIV RNA load and drug resistance testing and monitoring due to lack of experience of health professionals, and weak infrastructure and health care system contribute to delay in diagnosis of treatment failure	5
41	Telele *et al.,* 2018	Article	• The high rate of transmitted and preexisting drug resistance mutations in Ethiopian patients are identified	5

Note: Objective representation of the agreed thematic areas, 1 = Determine the prevalence and incidence of HIV and mortality rate in the City; 2 = Identify the hot spot areas in the City; 3 = Establish factors involved in driving the epidemic in the city, through analysis of behavioural, biological, socio-economic and demographic data; 4 = Identify most-at-risk and priority population groups in the City Administration (sex workers, in-school youth, prisoners/inmates, discordant couples and IDUs); 5 = Quickly assess service availability, access and utilization for the identified most at risk/priority populations groups in the City Administration

**Table 2:** HIV prevalence in Addis Ababa from EDHS and EPHIA [2, 3, 5, 6]

Studies	% HIV prevalence		
	Total	Women	Men
EDHS 2005	4.7	6.1	3.0
EDHS 2011	5.2	6.0	4.3
EDHS 2016	3.4	4.2	2.2
EPHIA 2017	3.1	–	–

## Results

### Study characteristics

The study includes characteristics such as the documents selected to be used in addressing the objectives and the findings in each document. The information relevant to the study in 41 documents was extracted and used accordingly as qualitative information as summarized in Table 1, and quantitative in Table 2 and Figs. 2, 3, 4, 5, 6.

### HIV prevalences

Surveys and assessment conducted in Addis Ababa such as EDHS [2, 5, 6], and EPHIA assessment [3] showed that prevalence of HIV is 4.7, 5.2, 3.4 and 3.1%, respectively (Table 2). Around 104,851 PLHIV live in Addis Ababa contributing nearly to 17.7% of the PLHIV population in the country, while it contributes 3.5% to the total population of the country [1].

Prevalences of HIV in Addis Ababa from Antenatal care (ANC) based surveillance of 2005–2014 are in a range of lowest in 2012 (4.4%) to the highest in 2005 (12.1%). The prevalence is relatively higher in 2014 (5.5%) than the prevalence in 2012 (4.4%). In addition, the prevalence from prevention of mother to child (PMTCT) surveillance report of 2016 (1.8%) is lower than the prevalence from ANC surveillance report of 2014 (Fig. 2).

### Hotspot areas of HIV transmission

The most common hot spots in Addis Ababa are areas where bars, groceries, pensions, guest houses, hotels, brothels, massage houses, khat houses, shisha houses, night clubs, drinking establishments and tourist frequented settings are concentrated. Condominiums are also mentioned as hotspot areas because sex workers commonly rent condos and are becoming centre of sexual transactions. There are various behavioural, biological and socio-economic predisposing risk factors that drive the epidemic in these hotspot areas in particular and the general population in general [13, 14].

### Factors involved in driving the epidemic

#### Behavioural factors

Low comprehensive knowledge about HIV/AIDS; alcohol and khat, shisha, substance abuse; gender based violence including rape; sex with multiple partners; practices of unsafe sex and inconsistent condom use; and dissatisfaction with sexual life in marriage are among major predisposing behavioural risk factors for the spread of HIV [13, 15, 16]. According to study conducted by OSSHD 72.5% of the intraveinous drug users (IDUs) in Addis Ababa had the habit of reusing needle and syringe [17]. In addition, early sexual debut, peer influence of young girls to engage in transactional sex, virginity selling, unfaithfulness for marriage, and boyfriend/girlfriend sharing are identified as risk factors for HIV transmission [13, 15, 18]. In other studies, the percentage of men who had sex with non-marital, non-cohabiting partners is highest in Addis Ababa (26%) compared to national (16%). In Addis Ababa, the highest mean number of lifetime sexual partners reported by men is 5.2; and 72.4% of women and 41.8% men reported using condom during last sexual intercourse with non-regular partner [2].

#### Biological factors

Discordant couples have the highest risk of acquiring HIV. From the total HIV positive couples in Addis Ababa, 4.3% of them were found to be discordant [2]. The proportion of disclosure of HIV/AIDS diagnosis to HIV-infected children is low. Almost one in ten HIV exposed infants become HIV positive in Ethiopia. Two and 4 % of the HIV exposed babies were HIV positive by 6 and 18 months, respectively [19]. There is low utilization of timely early infant diagnosis (EID) services. Being from the rural residence, home delivery, lack of understanding of the efficacy of ART, negative religious influences, and mixed infant feeding practices increased the risk of HIV transmission to children [20, 21].

#### Socio-economic factors

There are various socioeconomic factors contributing for high HIV epidemic in the City. High concentration of FSWs as means of livelihood; low socio-economic status; increasing sexual practices in massage houses; practice of intergenerational sex; high number of establishment like bars, hotels, restaurants, pastries, day and night clubs, brothels, pensions, local drink houses, and guest houses; engagement of gate-keepers, brokers and hotel owners in facilitating young girls to have transactional sex; growing number of construction and industry sites leading to increasing daily laborers from all parts of the country; living in groups to share house rent; high presence of movie houses that show pornographies; virtual appointments for dating and sexual relation; presence of naked dancing and call girls service; serving of the cosmetic and cloth shops for drugs distribution; increasing number of migration and visitors; cultural change and moral deterioration are the socio-economoc predisposing risk factors. Similarly, absence of recreational centers for youth, divorce and widowhood are aggravating factors for the spread of HIV in the City [2, 13, 22].

### Key and priority populations

MARPs Survey showed that the prevalence of HIV infection were 23% in self-identifying FSWs and 4.5% in truck drivers [23]; 4.2% in prison settings [24] and 5.7% HIV among mobile workers [14]. About 15.5% of drivers have misconceptions about HIV prevention methods [23]. According to recent estimates, there are about 200,000 FSWs in Ethiopia [25]. The majority of FSWs (57.5%) are 24 years and younger, and about 14% are 19 years or younger [26]. MARPs study [23] also showed that the size of FSWs in Addis Ababa was estimated to be 10,267. HIV prevalence in FSWs is four times higher than the general population.

A total of 4068 IDUs are estimated to be located in Addis Ababa [27]. The majority (72.5%) of the IDUs from Addis Ababa had the habit of reusing needle and syringe. Of the 177 Addis Ababa residents who claimed to have tested for HIV, 70 (39.5%) disclosed as HIV positive [17]. In addition, the prevalence of HIV among IDUs in Addis Ababa is 6, and 40% of IDUs reported ever-sharing needles. Furthermore, among HIV-positive IDUs, 60% reported sharing a needle the last time they injected [27]. Male IDUs are higher in number than female users at a ratio of 9:1 and 3/4 of the IDUs were below the age of 35 years [17].

In Addis Ababa, following on identifying FSWs as KP, various priority populations were also identified. The priority populations (PPs) are divorced and widowed persons; HIV-negative partners in discordant couples; long-distance truckers and taxi drivers and their assistants; paying clients and non-paying (‘Balukas’) of sex workers; individual engaged in transactional sex including sugar daddies and mummies, and waitresses; daily labourers in constructions and factories; IDUs; brokers, managers and workers in bars, groceries, pensions, guest houses, hotels, local drink houses, massage houses and shisha houses; and vulnerable adolescents and youth (immigrants from all parts of the country, migration returnees, house maids, street children, high education institution students and night school students) [13, 15, 25, 28].

### HIV transmission interventions

#### Behavioral interventions

Behavioural change communication (BCC), conducting peer and outreach education sessions, transmitting messages using mini-media and mass-media, condom promotion, and life skill trainings are the common behavioural interventions. The national average performance of condom distribution to MARPs group is 43.9% of the plan while for Addis Ababa it was 28.9% of their plan that is far below the national average. Likewise, the proportion of condom distributed to MARPs is very low, only 18.4% of the total condom distributed in the city [29, 30, Fig. 3].

#### Structural interventions

Structural interventions aiming to reduce vulnerability or ensuring service accessibility are being implemented including provision of economic strengthening, mapping and identification of hotspot areas and risky target groups, drop-in-centres (DICs), gender based violence and referral linkage [11, 29]. Findings indicated that economic strengthening interventions are diminishing in scale (Fig. 4).

#### Biomedical interventions

Biomedical interventions services are distribution of condom, HIV testing, sexually transmitted infection (STI) screening and treatment, ART, PMTCT and family planning, and ART post-exposure prophylaxis. In addition, ART pre-exposure prophylaxis for FSWs and discordant couples is at piloting stage. More than 10% of the BCC beneficiaries/FSWs had never been tested for HIV [26]. Some parents are refusing to give consent for their children to access HIV testing services (HTS) and ART services [30].

Behavioral, socio-economic and biomedical factors contributed to discontinuation ART. Heavy pill burden, fear of stigma and discrimination, cost and access to transportation, medication side effects, economic problems in the household, long travel due to distance from ART clinics, long waiting times, alcohol drinking, smoking, being with baseline CD4 < 200 cells/mm^3^, having mental illness, being bed ridden functional status, and dissatisfaction with healthcare services were risk factors for ART discontinuation. Males were reported to be most affected by discontinuation from being away from home [31–34]. More than 6% of HIV positive FSWs who started ART reported discontinuation of treatment for more than seven days in the three months prior to the assessment [26]. With the introduction of appointment spacing, some patients complain of lack of storage space for the six-month supply of ARTs, poor storage conditions for their medicines, and preference of frequent follow up. On the other hand, health workers are also concerned about adherence given the less frequent contact of PLHIV with the health services [35].

The HIV care and treatment service coverage indicated ART coverage is 74.6%, and viral load testing coverage is about 60% with 87.5% viral suppression among those who received viral load testing [11]. The national average for the first, second and third 90’s for urban Ethiopia is 72, 71 and 70.1%, respectively. VLS among 15–64 years of age HIV-positives in urban areas is close to the target (70.1%) but varies by age, sex and region. VLS is distinctly lower at 48.2% in youth 15–24 compared to the adult above 25 years of age. The status of the three 90’s for Addis Ababa is below the national urban average. Status of the three 90’s in Addis Ababa for the age group 0–64 years is lower than the national average which is: 65.2% for the 1st 90, 63.3% for the 2nd 90 and 58.2% of all PLHIV had VLS with viral load level of < 1000 copies/ml [3]. In Addis Ababa, the total number of clients on ART were 94,240 and 3616 were newly enrolled during the reporting period. The retention at 12 months was 87% [29, Fig. 5].

The Addis Ababa Mortality Surveillance Program using burial surveillance with verbal autopsy method [12] to identify AIDS and other causes of death showed that HIV/AIDS mortality is higher among females (12.1%) as compared to males (9.5%). In Addis Ababa from 2007 to 2010, an overall declining trends of AIDS related mortality was observed. However, starting 2010 onwards it seems stabilized (Fig. 6).

## Discussion

The current HIV program is applying targeted approaches so that both national and global targets are realized. In cognizant with that, Ethiopia has identified KPPs based on prevalence rate of specific groups and its respective context. The recognized KP group in Ethiopia is the FSWs. FSWs in Ethiopia are identified as KP in response to HIV epidemic as they are highly and consistently exposed to risky sexual practices which lead to HIV infection and transmission. Hence, evidence show that HIV prevalence among FSWs is the highest compared to other risk groups. The density of the FSWs population correlates closely with high PLHIV burden [15, 28, 36]. HIV transmission interventions include behavioural, biomedical and structural components. The current strategic plan intends to achieve the three 90 targets by 2020 through targeted social mobilization and HIV testing, linkage to care, quality of HIV treatment, and virtual elimination of MTCT, envisioning ending AIDS by 2030 [37].

There is relative decline of HIV prevalence from 2005 and 2016 EDHS findings in the city. However, antenatal clinic (ANC) based surveillance 2014 (5.5%), and PMTCT 2016 (1.8%) HIV prevalence [7] revealed that there is significant variation in reduction of HIV prevalence between the two study findings. Therefore, the comparison of the ANC and PMTCT surveillances findings needs cautious interpretation. There is need of implementing different behavioural, structural and biomedical transmission intervention measures on behavioural, biological and socio-economic factors that predisposed to HIV/AIDS in order to prevent and control rapid transmission. In connection to relatively better economic activities and social services, Addis Ababa attracts productive age groups from all over the country. Besides, the City serves as a gateway to the world. This also implies huge cultural exchange and dynamic and complex sexual behaviour, practices and networking catalysed by the presence of various types of sex workers [13]. There is sexual mixing of KPPs with the general population. The most affected populations are diluted by the general population. These serve as factors impacting the HIV transmission which call for a need to innovative HIV prevention and control strategies. Therefore, targeted activities need to be considered in designing strategies to promote testing and tracing HIV positives. Prevention and control measures should also be strengthened in the general population as well.

Combination ART acheives sustained HIV viral suppression and contributes to improvement in the quality of life, and reductions in mortality, progression to AIDS, opportunistic infections (OIs), hospitalization, and decreased HIV transmission to uninfected persons [38]. The stability of death by HIV/AIDS in Addis Ababa based on the studies [12] may be explained by better adherence follow-up and access for care and treatment.

Noncompliance to medical instruction and poor adherence fosters emergence of drug resistance. In addition, limited availability of laboratory services such as HIV RNA load and drug resistance testing and monitoring due to lack of experience of health professionals, and weak infrastructure and health care system contribute to delay in diagnosis of treatment failure [39, 40]. The high rate of transmitted and preexisting drug resistance mutations in Ethiopian patients are identified [41]. The finding of HIV-positives with high viral load in some studies [3] alarms the presence of people with high viral load which increases the risk of HIV transmission in the community.

There are gaps and challenges of HIV/AIDS prevention and control in Addis Ababa. Some of the gaps and challenges are weak monitoring of the quality of interventions, less emphasis in prevention, limited linkage of positive clients, lost to follow up, long turnaround time of viral load (VL) and EID tests, limited index-case-testings, limited effort in preventing substance abuse, inconsistent supply of test kits and condom, financial shortage, limited manpower and coordination, data quality problem, and gap in use of program data or research findings. Therefore, those challenges should be solved in order to achieve the three 90’s.

Developing appropriate service package, implement targeted intervention emphasizing on primary prevention; update existing implementation manuals to address the current situations and emerging vulnerability factors; create awareness to and engage hotel, bar, night clubs, pension, etc. owners and managers and police officers to play a key role in HIV prevention; ensuring availability of condom for KPPs including during the night time; strengthen a mechanism to work with media; promote open discussion about sexual and reproductive health; ensure availability and accessibility of commodities like ARTs, test kits and condoms; strengthen targeted testing to enhance identification of new cases and linking them to care and treatment services; ensuring effective implementation of legal framework governing massage houses, drug uses, shisha and khat houses as well as illegal brokers and rehabilitation of IDU are required. In order to implement these recommendations, gaps in critical enablers such as social mobilization, coordination, political commitment, resource, partnership, monitoring and evaluation, and data quality have to be addressed.

The limitations of this systematic review were the bad data quality in most works, and limitations of the quantitative secondary information due to their own inherent design and personal errors. Collection of secondary quantitative data was tried for many variables; however, interpretable data were found only for presented variables. In addition, due to many documents excluded and done by myself only, there may be bias and a chance of missing relevant information to be analyzed.

## Conclusions

Generally, even if the HIV prevalence is stabilized, the prevalence varies along different groups of the population within socio-demographic factors. There are many behavioural, biological and socio-economic factors that predisposed to HIV/AIDS. In addition, behavioural, structural and biomedical transmission intervention mechanisms are also affected by finance and lack of skilled man-power. Therefore, implementing targeted intervention focusing on primary prevention; update existing manuals and materials to address the current situations; ensuring availability of ARTs, test kits and condoms; broadcast key HIV messages on selected outlets; promote open discussion about sexual and reproductive health; strengthen targeted testing through index-case-testing, case-based-surveillance and social-network-strategy (SNS) to reach undiagnosed and new HIV infected people and linking them to care and treatment services; ensuring legal framework of governing massage houses, drug uses, shisha and khat houses, as well as illegal brokers are recommended in order to solve these challenges and fill the gaps. If it is able to accomplish these activities with plan in collaboration with partners, there will be bright future prospect to accomplish the three 90’s as planned.

## Data Availability

This paper is a systematic review of previously published data. All data generated or analysed during this study are included in this published article.

## Abberviations

AAHAPCO: Addis Ababa HIV/AIDS Prevention and Control Office
AAHB: Addis Ababa Health Bureau.
ANC: Antenatal clinic
ART: Antiretroviral therapy
BCC: Behavioural change communication
CBS: Case based surveillance
CDC: Americal Centre for Diseases Control and Prevention.
CSA: Central Statistical Agency
DICs: Drop-in-centres
EDHS: Ethiopian demographic and health survey.
EFY: Ethiopian fiscal year
EID: Early infant diagnosis
EPHA: Ethiopian Public Health Association
EPHI: Ethiopian Public Health Institute
EPHIA: Ethiopian population based HIV impact assessment
FHAPCO: Federal HIV/AIDS Prevention and Control Office
FMOE: Federal Ministry of Education
FMOH: Federal Ministry of Health
FSWs: Female sex workers.
HAART: Highly active antiretroviral therapy
HTS: HIV testing services
ICT: Index case testing
IDUs: Intraveinous drug users
IGA: Income generating activities.
KP: Key population
KPP: Key and priority population
MARPs: Most at risk populations
MTCT: Mother-to-Child-Transmission
OIs: Opportunistic infections.
OSSHD: Organization for Social Services, Health and Development
PEPFAR: President’s Emergency Plan for AIDS Relief
PITC: Professional initiated testing and counseling
PLHIV: People Living With HIV
PMTCT: Prevention of Mother to Child transmission
PNS: Partner notofication services
PP: Priority population
PPs: Priority populations
PSI/E: Population Services International, Ethiopia
SNNP: Sothern Nations, Nationalities and Peoples
SNS: Social networking strategy
STI: Sexually transmitted infections
UNODC: United Nations Office on Drug and Crime
VL: Viral load
VLS: Viral load suppression
WHO: World Health Organization
